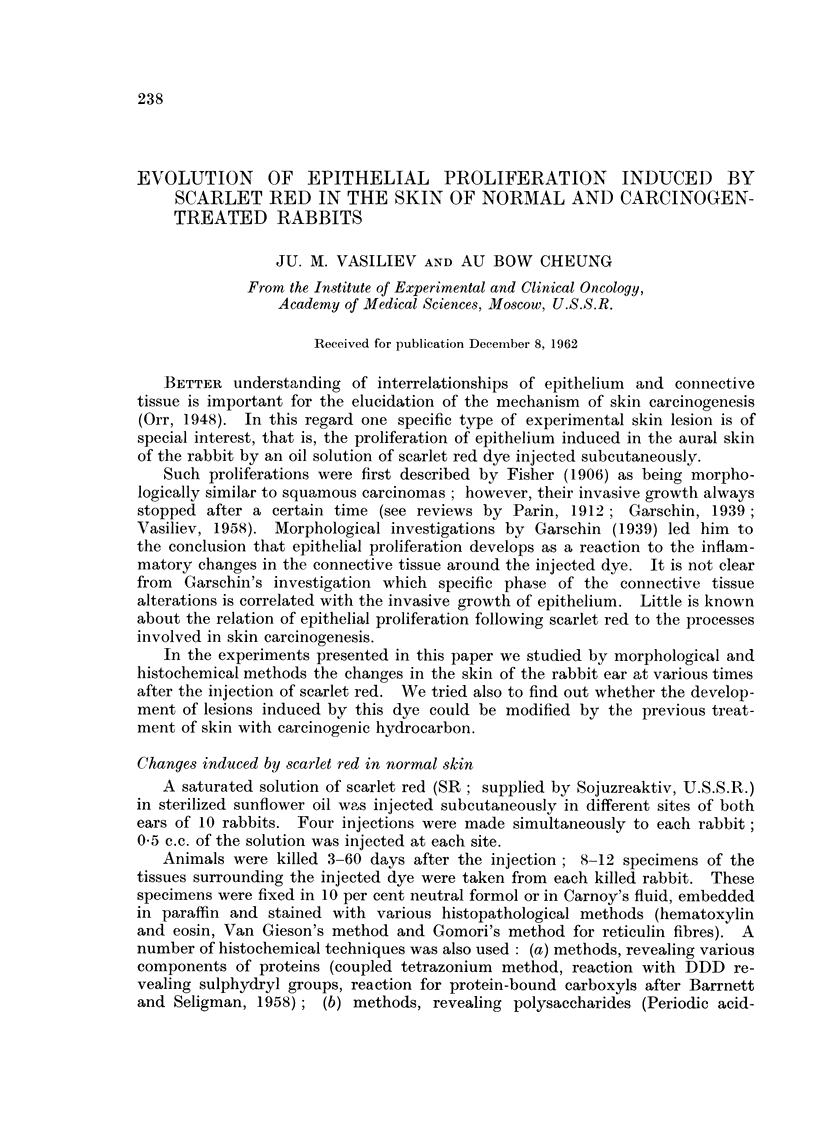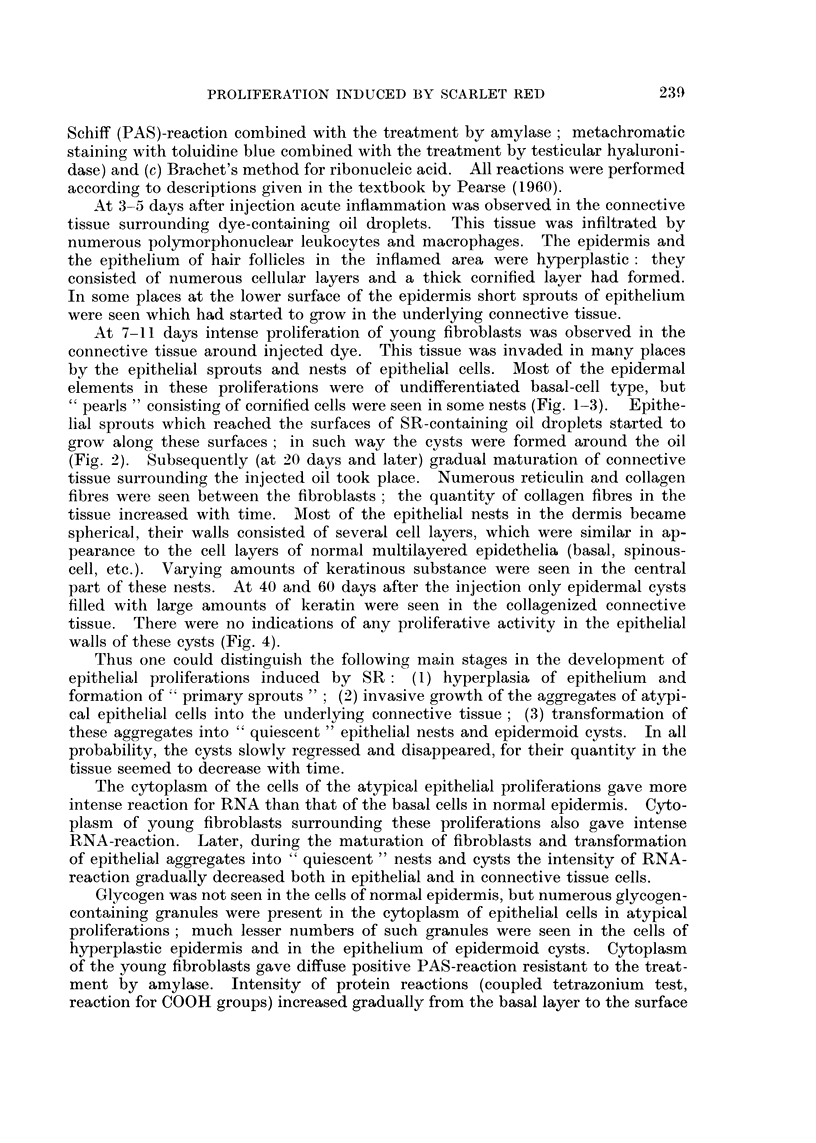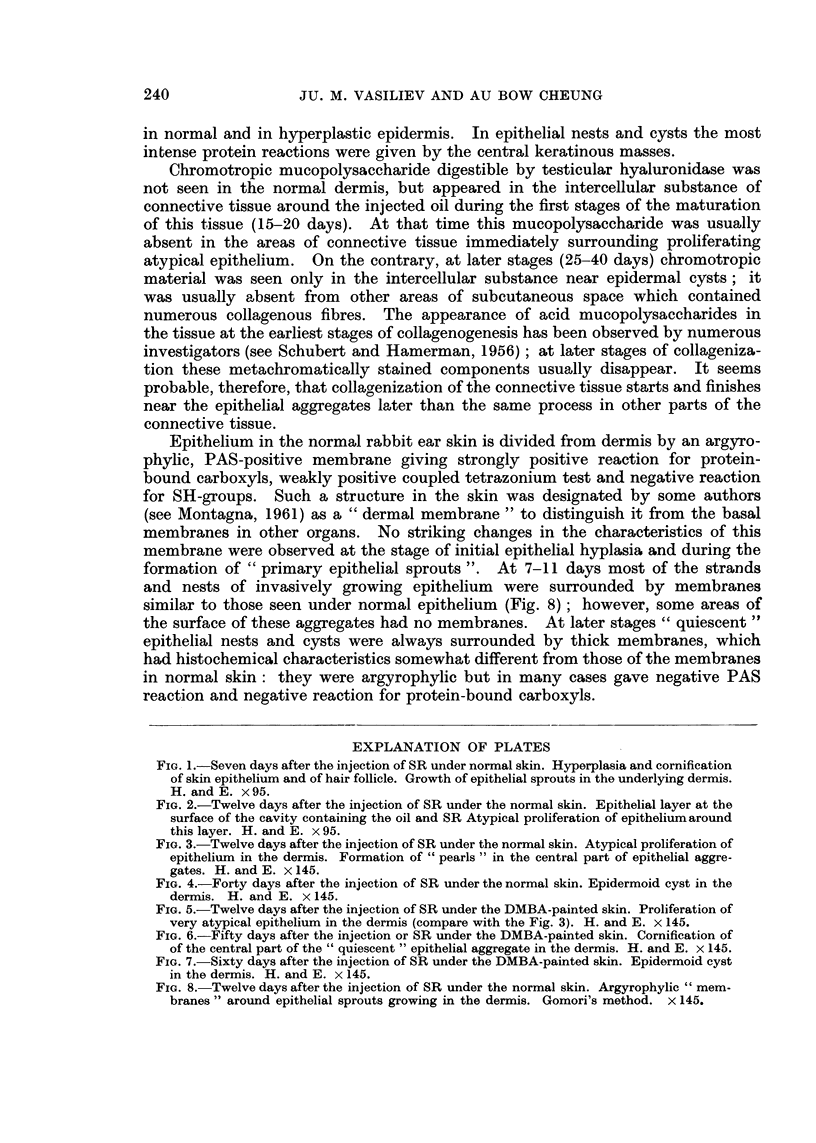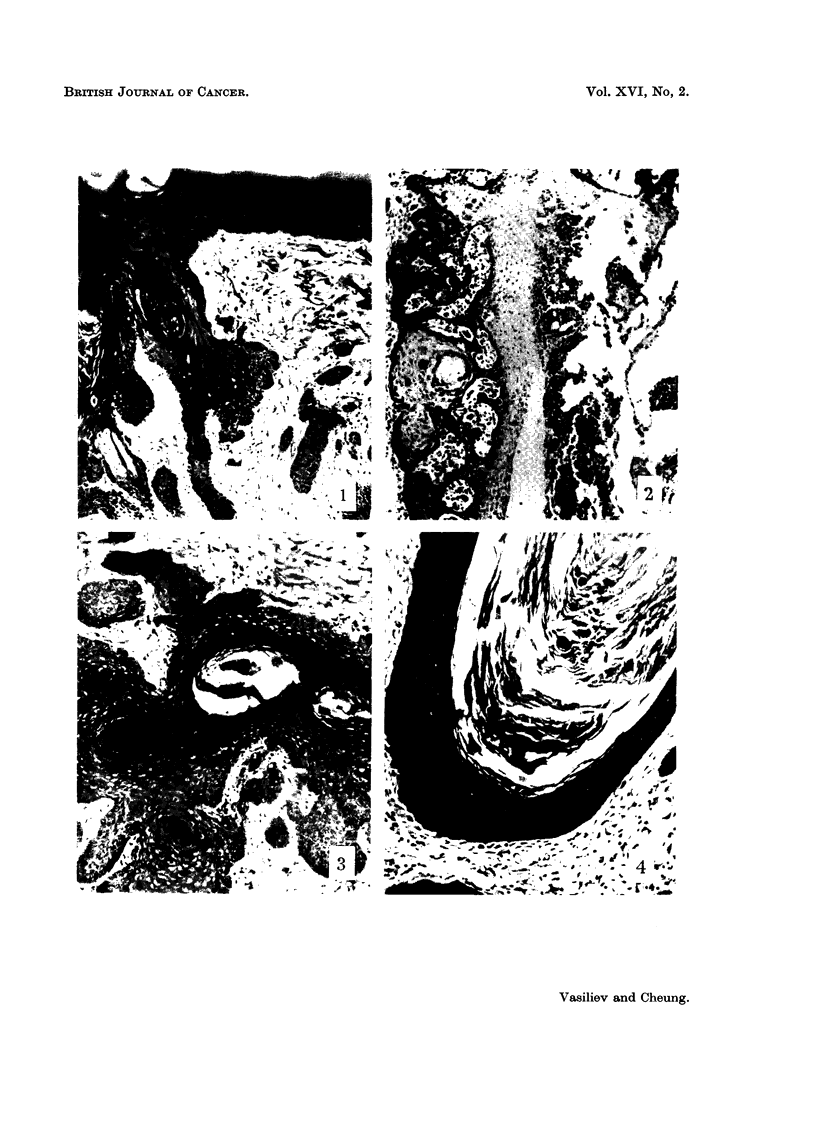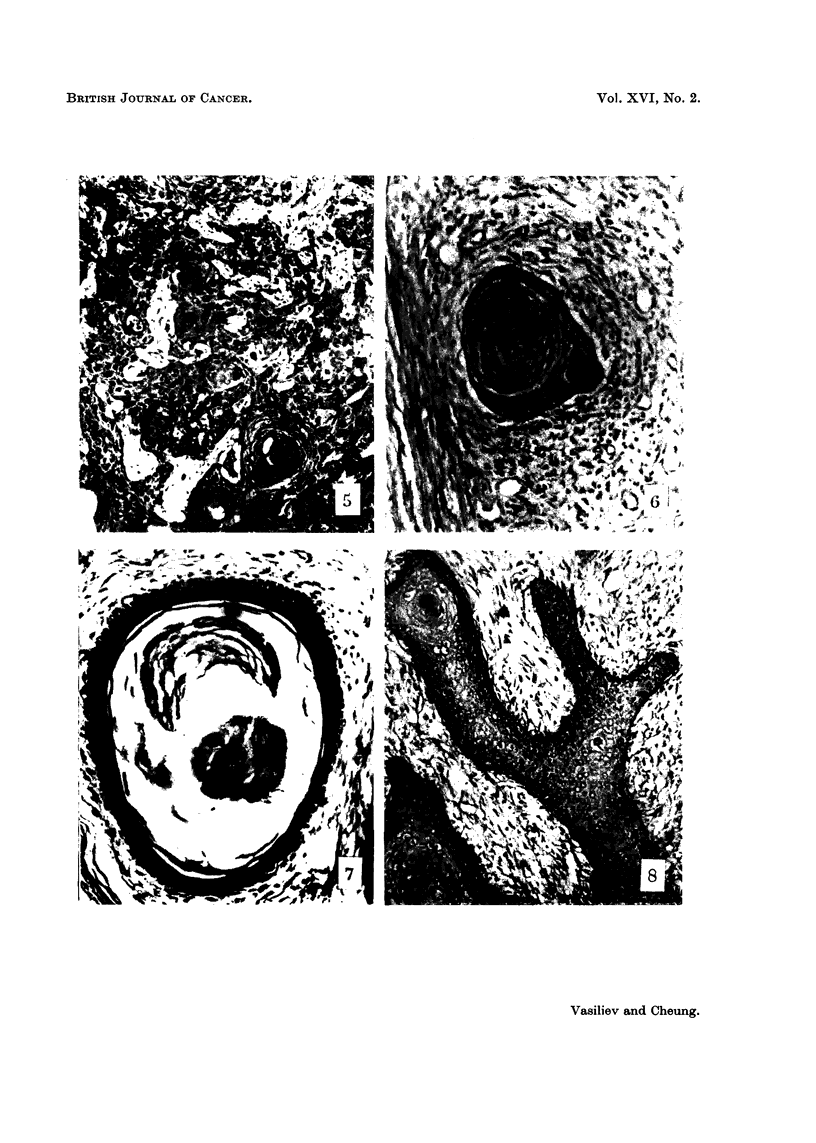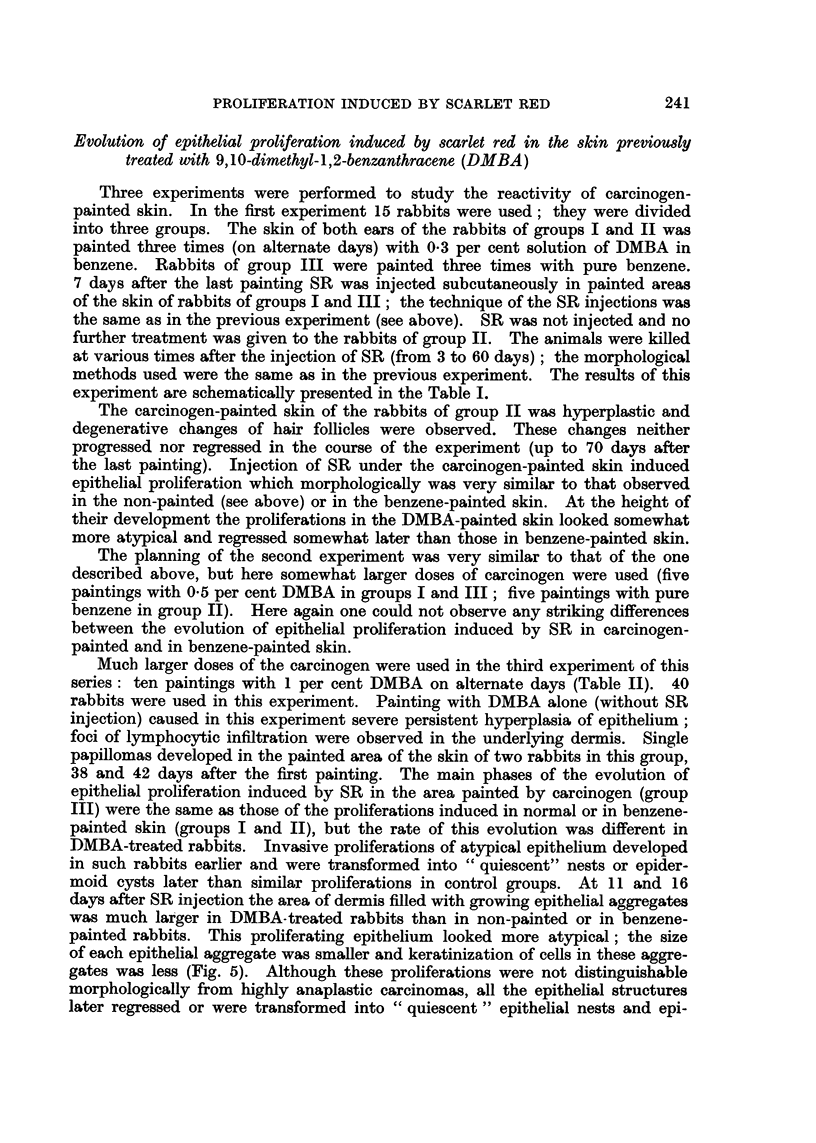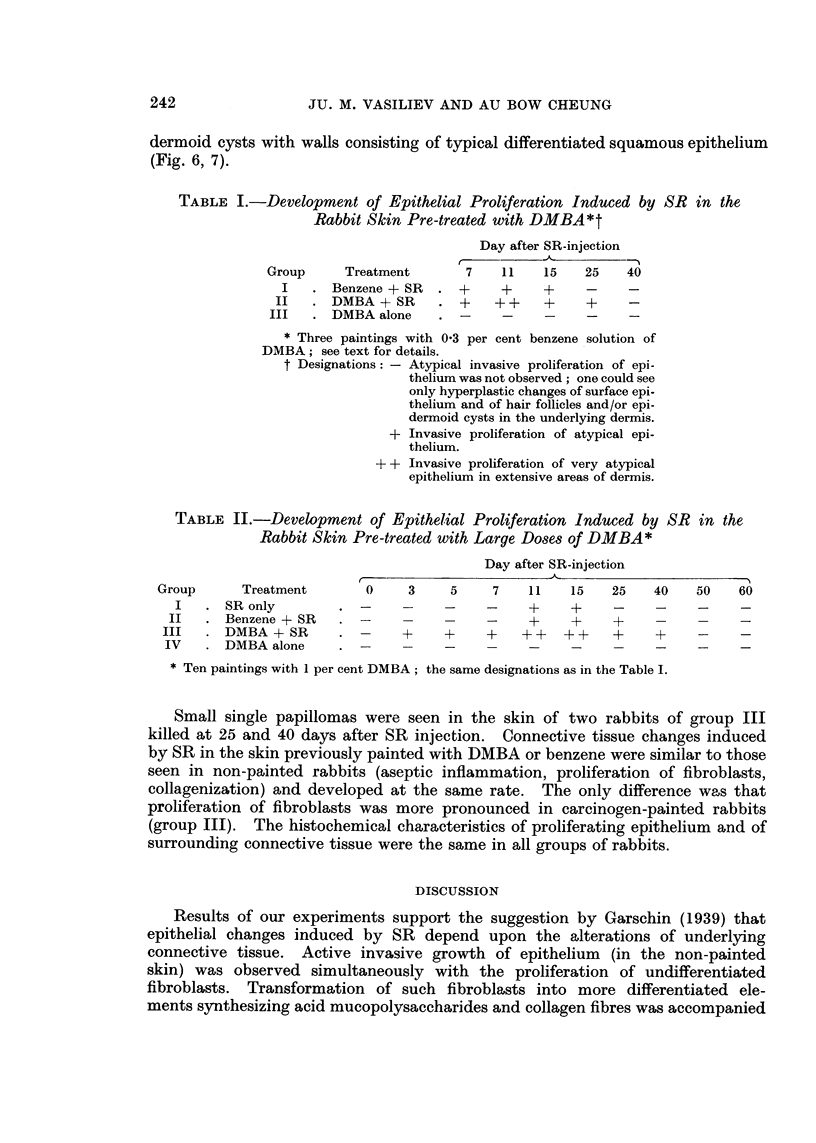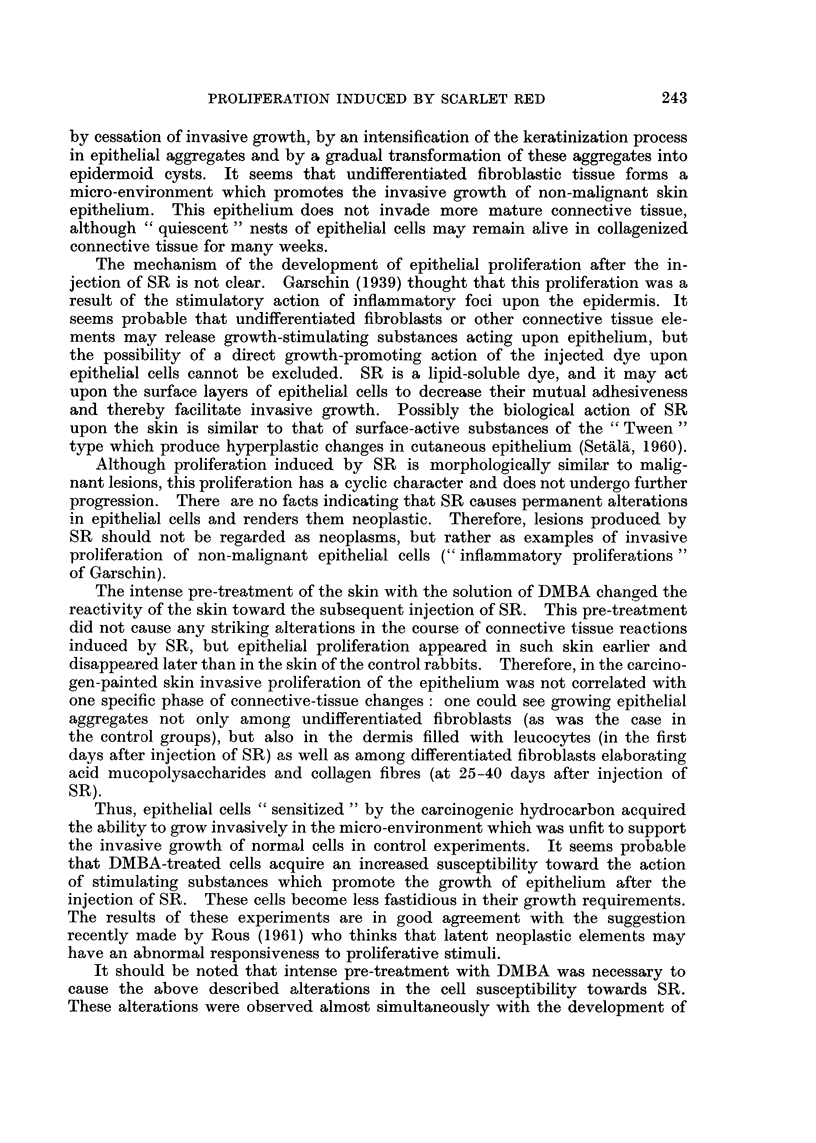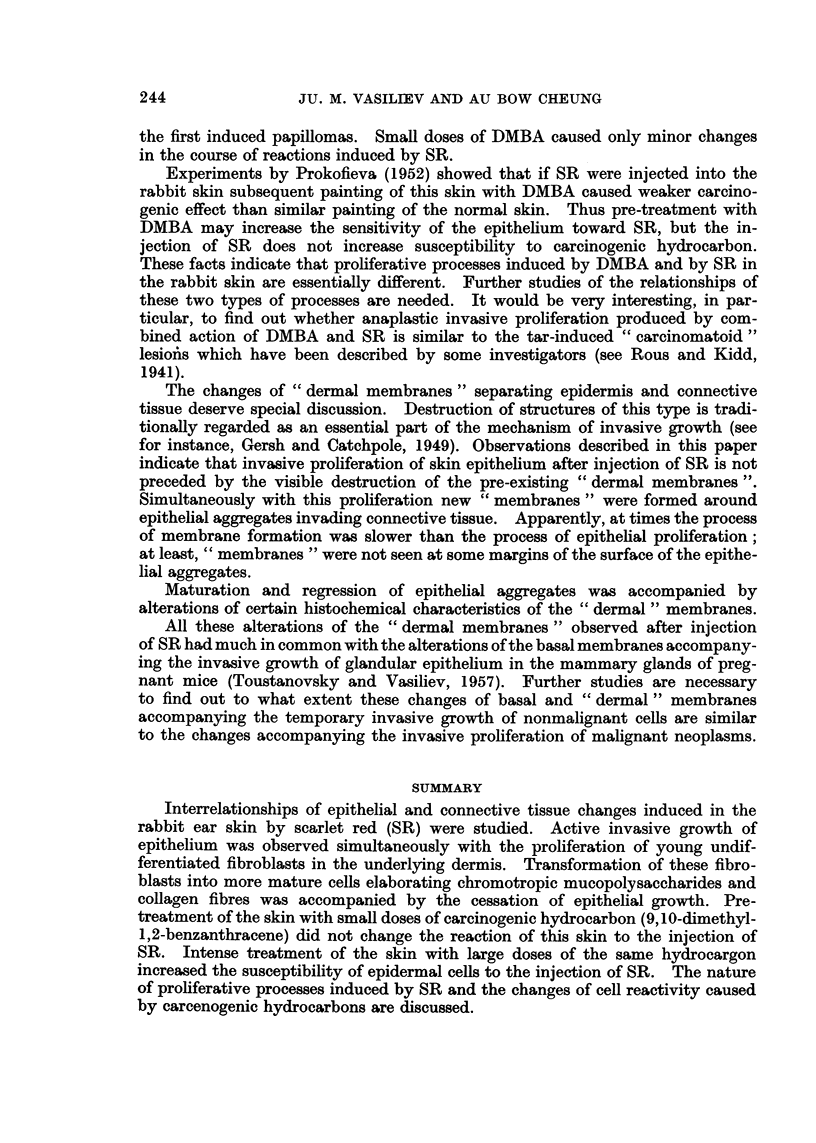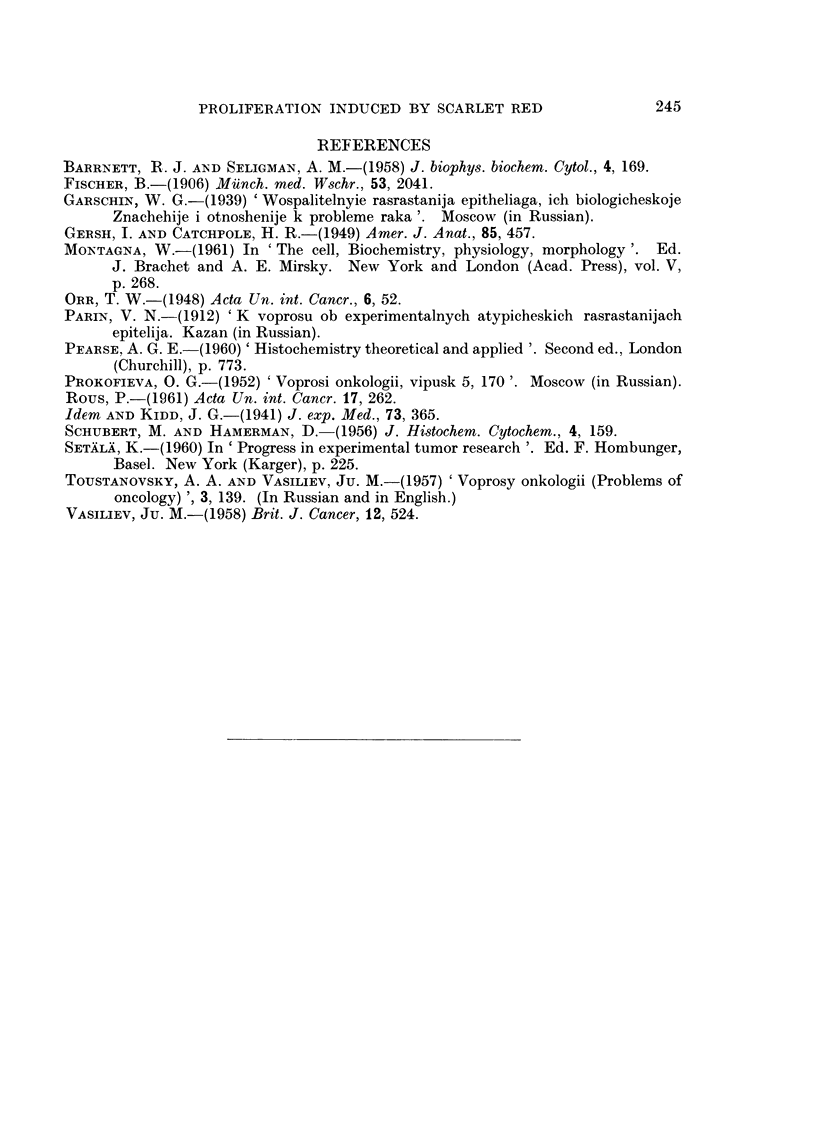# Evolution of Epithelial Proliferation induced by Scarlet Red in the Skin of Normal and Carcinogen Treated Rabbits

**DOI:** 10.1038/bjc.1962.27

**Published:** 1962-06

**Authors:** Ju. M. Vasiliev, Au Bow Cheung

## Abstract

**Images:**


### EVOLUTION OF EPITHELIAL PROLIFERATION INDUCED BY SCARLET RED IN THE SKIN OF NORMAL AND CARCINOGEN-TREATED RABBITS

JU. M. VASILIEV AND AU BOW CHEUNG

*From the Institute of Experimental and Clinical Oncology, Academy of Medical Sciences, Moscow, U.S.S.R.*

Received for publication December 8, 1962

BETTER understanding of interrelationships of epithelium and connective tissue is important for the elucidation of the mechanism of skin carcinogenesis (Orr, 1948). In this regard one specific type of experimental skin lesion is of special interest, that is, the proliferation of epithelium induced in the aural skin of the rabbit by an oil solution of scarlet red dye injected subcutaneously.

Such proliferations were first described by Fisher (1906) as being morphologically similar to squamous carcinomas ; however, their invasive growth always stopped after a certain time (see reviews by Parin, 1912 ; Garschin, 1939 ; Vasiliev, 1958). Morphological investigations by Garschin (1939) led him to the conclusion that epithelial proliferation develops as a reaction to the inflammatory changes in the connective tissue around the injected dye. It is not clear from Garschin's investigation which specific phase of the connective tissue alterations is correlated with the invasive growth of epithelium. Little is known about the relation of epithelial proliferation following scarlet red to the processes involved in skin carcinogenesis.

In the experiments presented in this paper we studied by morphological and histochemical methods the changes in the skin of the rabbit ear at various times after the injection of scarlet red. We tried also to find out whether the development of lesions induced by this dye could be modified by the previous treatment of skin with carcinogenic hydrocarbon.

*Changes induced by scarlet red in normal skin*

A saturated solution of scarlet red (SR ; supplied by Sojuzreaktiv, U.S.S.R.) in sterilized sunflower oil was injected subcutaneously in different sites of both ears of 10 rabbits. Four injections were made simultaneously to each rabbit ; 0·5 c.c. of the solution was injected at each site.

Animals were killed 3–60 days after the injection ; 8–12 specimens of the tissues surrounding the injected dye were taken from each killed rabbit. These specimens were fixed in 10 per cent neutral formol or in Carnoy's fluid, embedded in paraffin and stained with various histopathological methods (hematoxylin and eosin, Van Gieson's method and Gomori's method for reticulin fibres). A number of histochemical techniques was also used : (*a*) methods, revealing various components of proteins (coupled tetrazonium method, reaction with DDD revealing sulphydryl groups, reaction for protein-bound carboxyls after Barrnett and Seligman, 1958) ; (*b*) methods, revealing polysaccharides (Periodic acid-

Schiff (PAS)-reaction combined with the treatment by amylase ; metachromatic staining with toluidine blue combined with the treatment by testicular hyaluronidase) and (c) Brachet's method for ribonucleic acid. All reactions were performed according to descriptions given in the textbook by Pearse (1960).

At 3–5 days after injection acute inflammation was observed in the connective tissue surrounding dye-containing oil droplets. This tissue was infiltrated by numerous polymorphonuclear leukocytes and macrophages. The epidermis and the epithelium of hair follicles in the inflamed area were hyperplastic : they consisted of numerous cellular layers and a thick cornified layer had formed. In some places at the lower surface of the epidermis short sprouts of epithelium were seen which had started to grow in the underlying connective tissue.

At 7–11 days intense proliferation of young fibroblasts was observed in the connective tissue around injected dye. This tissue was invaded in many places by the epithelial sprouts and nests of epithelial cells. Most of the epidermal elements in these proliferations were of undifferentiated basal-cell type, but " pearls " consisting of cornified cells were seen in some nests (Fig. 1–3). Epithelial sprouts which reached the surfaces of SR-containing oil droplets started to grow along these surfaces ; in such way the cysts were formed around the oil (Fig. 2). Subsequently (at 20 days and later) gradual maturation of connective tissue surrounding the injected oil took place. Numerous reticulin and collagen fibres were seen between the fibroblasts ; the quantity of collagen fibres in the tissue increased with time. Most of the epithelial nests in the dermis became spherical, their walls consisted of several cell layers, which were similar in appearance to the cell layers of normal multilayered epidethelia (basal, spinous-cell, etc.). Varying amounts of keratinous substance were seen in the central part of these nests. At 40 and 60 days after the injection only epidermal cysts filled with large amounts of keratin were seen in the collagenized connective tissue. There were no indications of any proliferative activity in the epithelial walls of these cysts (Fig. 4).

Thus one could distinguish the following main stages in the development of epithelial proliferations induced by SR : (1) hyperplasia of epithelium and formation of " primary sprouts " ; (2) invasive growth of the aggregates of atypical epithelial cells into the underlying connective tissue ; (3) transformation of these aggregates into " quiescent " epithelial nests and epidermoid cysts. In all probability, the cysts slowly regressed and disappeared, for their quantity in the tissue seemed to decrease with time.

The cytoplasm of the cells of the atypical epithelial proliferations gave more intense reaction for RNA than that of the basal cells in normal epidermis. Cytoplasm of young fibroblasts surrounding these proliferations also gave intense RNA-reaction. Later, during the maturation of fibroblasts and transformation of epithelial aggregates into " quiescent " nests and cysts the intensity of RNA-reaction gradually decreased both in epithelial and in connective tissue cells.

Glycogen was not seen in the cells of normal epidermis, but numerous glycogen-containing granules were present in the cytoplasm of epithelial cells in atypical proliferations ; much lesser numbers of such granules were seen in the cells of hyperplastic epidermis and in the epithelium of epidermoid cysts. Cytoplasm of the young fibroblasts gave diffuse positive PAS-reaction resistant to the treatment by amylase. Intensity of protein reactions (coupled tetrazonium test, reaction for COOH groups) increased gradually from the basal layer to the surface in normal and in hyperplastic epidermis. In epithelial nests and cysts the most intense protein reactions were given by the central keratinous masses.

Chromotropic mucopolysaccharide digestible by testicular hyaluronidase was not seen in the normal dermis, but appeared in the intercellular substance of connective tissue around the injected oil during the first stages of the maturation of this tissue (15–20 days). At that time this mucopolysaccharide was usually absent in the areas of connective tissue immediately surrounding proliferating atypical epithelium. On the contrary, at later stages (25–40 days) chromotropic material was seen only in the intercellular substance near epidermal cysts ; it was usually absent from other areas of subcutaneous space which contained numerous collagenous fibres. The appearance of acid mucopolysaccharides in the tissue at the earliest stages of collagenogenesis has been observed by numerous investigators (see Schubert and Hamerman, 1956) ; at later stages of collagenization these metachromatically stained components usually disappear. It seems probable, therefore, that collagenization of the connective tissue starts and finishes near the epithelial aggregates later than the same process in other parts of the connective tissue.

Epithelium in the normal rabbit ear skin is divided from dermis by an argyrophylic, PAS-positive membrane giving strongly positive reaction for protein-bound carboxyls, weakly positive coupled tetrazonium test and negative reaction for SH-groups. Such a structure in the skin was designated by some authors (see Montagna, 1961) as a " dermal membrane " to distinguish it from the basal membranes in other organs. No striking changes in the characteristics of this membrane were observed at the stage of initial epithelial hyplasia and during the formation of " primary epithelial sprouts ". At 7–11 days most of the strands and nests of invasively growing epithelium were surrounded by membranes similar to those seen under normal epithelium (Fig. 8) ; however, some areas of the surface of these aggregates had no membranes. At later stages " quiescent " epithelial nests and cysts were always surrounded by thick membranes, which had histochemical characteristics somewhat different from those of the membranes in normal skin : they were argyrophylic but in many cases gave negative PAS reaction and negative reaction for protein-bound carboxyls.

---

### EXPLANATION OF PLATES

FIG. 1.—Seven days after the injection of SR under normal skin. Hyperplasia and cornification of skin epithelium and of hair follicle. Growth of epithelial sprouts in the underlying dermis. H. and E. ×95.

FIG. 2.—Twelve days after the injection of SR under the normal skin. Epithelial layer at the surface of the cavity containing the oil and SR Atypical proliferation of epithelium around this layer. H. and E. ×95.

FIG. 3.—Twelve days after the injection of SR under the normal skin. Atypical proliferation of epithelium in the dermis. Formation of " pearls " in the central part of epithelial aggregates. H. and E. ×145.

FIG. 4.—Forty days after the injection of SR under the normal skin. Epidermoid cyst in the dermis. H. and E. ×145.

FIG. 5.—Twelve days after the injection of SR under the DMBA-painted skin. Proliferation of very atypical epithelium in the dermis (compare with the Fig. 3). H. and E. ×145.

FIG. 6.—Fifty days after the injection or SR under the DMBA-painted skin. Cornification of of the central part of the " quiescent " epithelial aggregate in the dermis. H. and E. ×145.

FIG. 7.—Sixty days after the injection of SR under the DMBA-painted skin. Epidermoid cyst in the dermis. H. and E. ×145.

FIG. 8.—Twelve days after the injection of SR under the normal skin. Argyrophylic " membranes " around epithelial sprouts growing in the dermis. Gomori's method. ×145.

Vasiliev and Cheung.

Vasiliev and Cheung.

*Evolution of epithelial proliferation induced by scarlet red in the skin previously treated with* 9,10-*dimethyl*-1,2-*benzanthracene* (*DMBA*)

Three experiments were performed to study the reactivity of carcinogen-painted skin. In the first experiment 15 rabbits were used ; they were divided into three groups. The skin of both ears of the rabbits of groups I and II was painted three times (on alternate days) with 0·3 per cent solution of DMBA in benzene. Rabbits of group III were painted three times with pure benzene. 7 days after the last painting SR was injected subcutaneously in painted areas of the skin of rabbits of groups I and III ; the technique of the SR injections was the same as in the previous experiment (see above). SR was not injected and no further treatment was given to the rabbits of group II. The animals were killed at various times after the injection of SR (from 3 to 60 days) ; the morphological methods used were the same as in the previous experiment. The results of this experiment are schematically presented in the Table I.

The carcinogen-painted skin of the rabbits of group II was hyperplastic and degenerative changes of hair follicles were observed. These changes neither progressed nor regressed in the course of the experiment (up to 70 days after the last painting). Injection of SR under the carcinogen-painted skin induced epithelial proliferation which morphologically was very similar to that observed in the non-painted (see above) or in the benzene-painted skin. At the height of their development the proliferations in the DMBA-painted skin looked somewhat more atypical and regressed somewhat later than those in benzene-painted skin.

The planning of the second experiment was very similar to that of the one described above, but here somewhat larger doses of carcinogen were used (five paintings with 0·5 per cent DMBA in groups I and III ; five paintings with pure benzene in group II). Here again one could not observe any striking differences between the evolution of epithelial proliferation induced by SR in carcinogen-painted and in benzene-painted skin.

Much larger doses of the carcinogen were used in the third experiment of this series : ten paintings with 1 per cent DMBA on alternate days (Table II). 40 rabbits were used in this experiment. Painting with DMBA alone (without SR injection) caused in this experiment severe persistent hyperplasia of epithelium ; foci of lymphocytic infiltration were observed in the underlying dermis. Single papillomas developed in the painted area of the skin of two rabbits in this group, 38 and 42 days after the first painting. The main phases of the evolution of epithelial proliferation induced by SR in the area painted by carcinogen (group III) were the same as those of the proliferations induced in normal or in benzene-painted skin (groups I and II), but the rate of this evolution was different in DMBA-treated rabbits. Invasive proliferations of atypical epithelium developed in such rabbits earlier and were transformed into " quiescent" nests or epidermoid cysts later than similar proliferations in control groups. At 11 and 16 days after SR injection the area of dermis filled with growing epithelial aggregates was much larger in DMBA-treated rabbits than in non-painted or in benzene-painted rabbits. This proliferating epithelium looked more atypical ; the size of each epithelial aggregate was smaller and keratinization of cells in these aggregates was less (Fig. 5). Although these proliferations were not distinguishable morphologically from highly anaplastic carcinomas, all the epithelial structures later regressed or were transformed into " quiescent " epithelial nests and epidermoid cysts with walls consisting of typical differentiated squamous epithelium (Fig. 6, 7).

TABLE I.—*Development of Epithelial Proliferation Induced by SR in the Rabbit Skin Pre-treated with DMBA**†

| Group | Treatment | Day after SR-injection 7 | 11 | 15 | 25 | 40 |
|---|---|---|---|---|---|---|
| I | Benzene + SR | + | + | + | – | – |
| II | DMBA + SR | + | ++ | + | + | – |
| III | DMBA alone | – | – | – | – | – |

* Three paintings with 0·3 per cent benzene solution of DMBA ; see text for details.

† Designations : – Atypical invasive proliferation of epithelium was not observed ; one could see only hyperplastic changes of surface epithelium and of hair follicles and/or epidermoid cysts in the underlying dermis.
+ Invasive proliferation of atypical epithelium.
++ Invasive proliferation of very atypical epithelium in extensive areas of dermis.

TABLE II.—*Development of Epithelial Proliferation Induced by SR in the Rabbit Skin Pre-treated with Large Doses of DMBA**

| Group | Treatment | Day after SR-injection 0 | 3 | 5 | 7 | 11 | 15 | 25 | 40 | 50 | 60 |
|---|---|---|---|---|---|---|---|---|---|---|---|
| I | SR only | – | – | – | – | + | + | – | – | – | – |
| II | Benzene + SR | – | – | – | – | + | + | + | – | – | – |
| III | DMBA + SR | – | + | + | + | ++ | ++ | + | + | – | – |
| IV | DMBA alone | – | – | – | – | – | – | – | – | – | – |

* Ten paintings with 1 per cent DMBA ; the same designations as in the Table I.

Small single papillomas were seen in the skin of two rabbits of group III killed at 25 and 40 days after SR injection. Connective tissue changes induced by SR in the skin previously painted with DMBA or benzene were similar to those seen in non-painted rabbits (aseptic inflammation, proliferation of fibroblasts, collagenization) and developed at the same rate. The only difference was that proliferation of fibroblasts was more pronounced in carcinogen-painted rabbits (group III). The histochemical characteristics of proliferating epithelium and of surrounding connective tissue were the same in all groups of rabbits.

### DISCUSSION

Results of our experiments support the suggestion by Garschin (1939) that epithelial changes induced by SR depend upon the alterations of underlying connective tissue. Active invasive growth of epithelium (in the non-painted skin) was observed simultaneously with the proliferation of undifferentiated fibroblasts. Transformation of such fibroblasts into more differentiated elements synthesizing acid mucopolysaccharides and collagen fibres was accompanied by cessation of invasive growth, by an intensification of the keratinization process in epithelial aggregates and by a gradual transformation of these aggregates into epidermoid cysts. It seems that undifferentiated fibroblastic tissue forms a micro-environment which promotes the invasive growth of non-malignant skin epithelium. This epithelium does not invade more mature connective tissue, although " quiescent " nests of epithelial cells may remain alive in collagenized connective tissue for many weeks.

The mechanism of the development of epithelial proliferation after the injection of SR is not clear. Garschin (1939) thought that this proliferation was a result of the stimulatory action of inflammatory foci upon the epidermis. It seems probable that undifferentiated fibroblasts or other connective tissue elements may release growth-stimulating substances acting upon epithelium, but the possibility of a direct growth-promoting action of the injected dye upon epithelial cells cannot be excluded. SR is a lipid-soluble dye, and it may act upon the surface layers of epithelial cells to decrease their mutual adhesiveness and thereby facilitate invasive growth. Possibly the biological action of SR upon the skin is similar to that of surface-active substances of the " Tween " type which produce hyperplastic changes in cutaneous epithelium (Setälä, 1960).

Although proliferation induced by SR is morphologically similar to malignant lesions, this proliferation has a cyclic character and does not undergo further progression. There are no facts indicating that SR causes permanent alterations in epithelial cells and renders them neoplastic. Therefore, lesions produced by SR should not be regarded as neoplasms, but rather as examples of invasive proliferation of non-malignant epithelial cells (" inflammatory proliferations " of Garschin).

The intense pre-treatment of the skin with the solution of DMBA changed the reactivity of the skin toward the subsequent injection of SR. This pre-treatment did not cause any striking alterations in the course of connective tissue reactions induced by SR, but epithelial proliferation appeared in such skin earlier and disappeared later than in the skin of the control rabbits. Therefore, in the carcinogen-painted skin invasive proliferation of the epithelium was not correlated with one specific phase of connective-tissue changes : one could see growing epithelial aggregates not only among undifferentiated fibroblasts (as was the case in the control groups), but also in the dermis filled with leucocytes (in the first days after injection of SR) as well as among differentiated fibroblasts elaborating acid mucopolysaccharides and collagen fibres (at 25–40 days after injection of SR).

Thus, epithelial cells " sensitized " by the carcinogenic hydrocarbon acquired the ability to grow invasively in the micro-environment which was unfit to support the invasive growth of normal cells in control experiments. It seems probable that DMBA-treated cells acquire an increased susceptibility toward the action of stimulating substances which promote the growth of epithelium after the injection of SR. These cells become less fastidious in their growth requirements. The results of these experiments are in good agreement with the suggestion recently made by Rous (1961) who thinks that latent neoplastic elements may have an abnormal responsiveness to proliferative stimuli.

It should be noted that intense pre-treatment with DMBA was necessary to cause the above described alterations in the cell susceptibility towards SR. These alterations were observed almost simultaneously with the development of the first induced papillomas. Small doses of DMBA caused only minor changes in the course of reactions induced by SR.

Experiments by Prokofieva (1952) showed that if SR were injected into the rabbit skin subsequent painting of this skin with DMBA caused weaker carcinogenic effect than similar painting of the normal skin. Thus pre-treatment with DMBA may increase the sensitivity of the epithelium toward SR, but the injection of SR does not increase susceptibility to carcinogenic hydrocarbon. These facts indicate that proliferative processes induced by DMBA and by SR in the rabbit skin are essentially different. Further studies of the relationships of these two types of processes are needed. It would be very interesting, in particular, to find out whether anaplastic invasive proliferation produced by combined action of DMBA and SR is similar to the tar-induced " carcinomatoid " lesions which have been described by some investigators (see Rous and Kidd, 1941).

The changes of " dermal membranes " separating epidermis and connective tissue deserve special discussion. Destruction of structures of this type is traditionally regarded as an essential part of the mechanism of invasive growth (see for instance, Gersh and Catchpole, 1949). Observations described in this paper indicate that invasive proliferation of skin epithelium after injection of SR is not preceded by the visible destruction of the pre-existing " dermal membranes ". Simultaneously with this proliferation new " membranes " were formed around epithelial aggregates invading connective tissue. Apparently, at times the process of membrane formation was slower than the process of epithelial proliferation ; at least, " membranes " were not seen at some margins of the surface of the epithelial aggregates.

Maturation and regression of epithelial aggregates was accompanied by alterations of certain histochemical characteristics of the " dermal " membranes.

All these alterations of the " dermal membranes " observed after injection of SR had much in common with the alterations of the basal membranes accompanying the invasive growth of glandular epithelium in the mammary glands of pregnant mice (Toustanovsky and Vasiliev, 1957). Further studies are necessary to find out to what extent these changes of basal and " dermal " membranes accompanying the temporary invasive growth of nonmalignant cells are similar to the changes accompanying the invasive proliferation of malignant neoplasms.

### SUMMARY

Interrelationships of epithelial and connective tissue changes induced in the rabbit ear skin by scarlet red (SR) were studied. Active invasive growth of epithelium was observed simultaneously with the proliferation of young undifferentiated fibroblasts in the underlying dermis. Transformation of these fibroblasts into more mature cells elaborating chromotropic mucopolysaccharides and collagen fibres was accompanied by the cessation of epithelial growth. Pretreatment of the skin with small doses of carcinogenic hydrocarbon (9,10-dimethyl-1,2-benzanthracene) did not change the reaction of this skin to the injection of SR. Intense treatment of the skin with large doses of the same hydrocargon increased the susceptibility of epidermal cells to the injection of SR. The nature of proliferative processes induced by SR and the changes of cell reactivity caused by carcenogenic hydrocarbons are discussed.

### REFERENCES

BARRNETT, R. J. AND SELIGMAN, A. M.—(1958) *J. biophys. biochem. Cytol.*, **4**, 169.
FISCHER, B.—(1906) *Münch. med. Wschr.*, **53**, 2041.
GARSCHIN, W. G.—(1939) ' Wospalitelnyie rasrastanija epitheliaga, ich biologicheskoje Znachehije i otnoshenije k probleme raka '. Moscow (in Russian).
GERSH, I. AND CATCHPOLE, H. R.—(1949) *Amer. J. Anat.*, **85**, 457.
MONTAGNA, W.—(1961) In ' The cell, Biochemistry, physiology, morphology '. Ed. J. Brachet and A. E. Mirsky. New York and London (Acad. Press), vol. V, p. 268.
ORR, T. W.—(1948) *Acta Un. int. Cancr.*, **6**, 52.
PARIN, V. N.—(1912) ' K voprosu ob experimentalnych atypicheskich rasrastanijach epitelija. Kazan (in Russian).
PEARSE, A. G. E.—(1960) ' Histochemistry theoretical and applied '. Second ed., London (Churchill), p. 773.
PROKOFIEVA, O. G.—(1952) ' Voprosi onkologii, vipusk 5, 170 '. Moscow (in Russian).
ROUS, P.—(1961) *Acta Un. int. Cancr.* **17**, 262.
*Idem* AND KIDD, J. G.—(1941) *J. exp. Med.*, **73**, 365.
SCHUBERT, M. AND HAMERMAN, D.—(1956) *J. Histochem. Cytochem.*, **4**, 159.
SETÄLÄ, K.—(1960) In ' Progress in experimental tumor research '. Ed. F. Hombunger, Basel. New York (Karger), p. 225.
TOUSTANOVSKY, A. A. AND VASILIEV, JU. M.—(1957) ' Voprosy onkologii (Problems of oncology) ', **3**, 139. (In Russian and in English.)
VASILIEV, JU. M.—(1958) *Brit. J. Cancer*, **12**, 524.